# The Use and Perceived Barriers of the Partograph at Public Health Institutions in East Gojjam Zone, Northwest Ethiopia

**DOI:** 10.29024/aogh.23

**Published:** 2018-04-30

**Authors:** Desalegne Zelellw, Teketo Tegegne

**Affiliations:** 1 School of Nursing, College of Medicine and Health Sciences, Bahir Dar University, ET; 2 College of Medicine and Health Sciences, Debre Markos University, ET

## Abstract

**Introduction:**

The partograph is a vital tool used to reduce maternal and fetal mortality and morbidity and to prevent prolonged and obstructed labor, as well as postpartum hemorrhage and fistula formation. This study explored the use and barriers of the partograph among obstetric caregivers in East Gojam Zone, Northwest Ethiopia.

**Methods and materials:**

A cross-sectional study design consisting of both quantitative and qualitative methods was utilized. Data was collected through a structured clinical observation checklist and semistructured questions. The content of the checklists was developed according to modified WHO partograph. Thematic analysis was employed using open code software version 3.6. Coding was done immediately after the data was collected. The coded data was defined and categorized into groups. Once the categories were identified and the names given, the data was related to the objectives of the study. Data was crosschecked to ensure consistency between the themes and the categories. Then we summarized the themes and drew conclusions that gave answers to the research questions.

**Results and conclusion:**

This study showed that participants believed partograph is an essential tool used to reduce maternal and fetal mortality and morbidity and to prevent prolonged and obstructed labor, as well as postpartum hemorrhage. They explained that work overload, lack of skill and competency, negligence, lack of motivation and a shortage of infrastructure and resources hindered utilization of the partograph.

## Introduction

The partograph is a standard tool used to improve maternal and perinatal outcomes [1]. It has been used since the 1950s in both developed and developing countries [2]. When it is used accurately and consistently, it can reduce maternal and perinatal morbidity and mortality due to prolonged labor, cephalopelvic disproportion, obstructed labor and neonatal asphyxia [3]. Preventing these outcomes required an adequate number of skilled health workers, especially midwives, with a positive attitude toward use of the partograph [1].

There are three forms of the partograph: the first is the original design that includes a latent phase of 8 hours, and the active phase starts at 3 cm of cervical dilation. The second form is a modified partograph, adapted in 2000 by the World Health Organization (WHO) for use in hospitals, that excludes the latent phase and starts the active phase at 4 cm of cervical dilation. The third form is simplified (further adapted) and used by skilled attendants in health centers; it excludes the latent phase and descent of the presenting part, and the active phase is color coded and starts at 4 cm of cervical dilation [4]. These serve as an early warning system and assist in early decision-making on transfer, augmentation and termination of labor [5].

It is evident that for effective use of the partograph obstetric caregivers should have comprehensive understanding of the functions of the partograph and should continuously monitor, document and interpret the collected information for early detection and prevention of maternal and neonatal complications [6]. Use of the partograph in relation to the innovations or changes that are made to it is impacted by caregivers’ difference in knowledge, attitude, awareness and confidence in its use; regular supportive supervision; quality assurance and organizational context [7].

In Ethiopia, lack of knowledge, negative attitudes, understaffing and lack of training were barriers to use of the partograph [8]. In addition, in Central Ethiopia, use of different monitoring tools, unavailability of tools, staff shortage and lack of trained caregivers were barriers to utilizing the partograph during labor [9]. The objective of this study was to assess the usage and perceived barriers of obstetric caregivers at public health institutions in East Gojjam Zone, Northwest Ethiopia.

## Methods and Materials

### Setting

The study was conducted at public health institutions from March to July 2015. East Gojjan Zone is comprised of 19 woredas, 101 health centers and 2 hospitals. There are 1,417 health professionals with qualifications of midwifery, nurse, public health, medical doctor or MSc in emergency surgery and obstetrics.

### Study design

Facility-based cross-sectional study design of both qualitative and quantitative methods were applied.

### Eligibility criteria

All health care providers who were working in government health facilities were included in the study. Pharmacy, laboratory and other health care providers who were not working in the labor and delivery room were excluded from the study.

### Data collection tools

Data was collected through focus group discussion (FGD) and observational checklists. Each focus group discussions consisted of eight to eleven members. Participants were encouraged to actively participate in the discussion. The facilitator directed the discussion in the right way. All information was recorded using a digital voice recorder, and notes were taken. The group members included nurses, midwives, public health officers and medical doctors. The majority of the members were nurses and midwives. The FGD discussion took sixty to niney minutes. The data was transcribed into Amharic language and then translated into English.

The checklist focused on the clinical skills of health care providers as related to labor, maternal conditions and fetal conditions. The checklist was developed from the modified WHO partograph, which consisted of ten items. Progress of labor uterine contraction, descent and cervical dilation were included. Fetal conditions, fetal heart rate, the molding of the skull and liquor were included, as well as maternal conditions, maternal pulse, maternal blood pressure, maternal temperature, urine volume, ketone and protein.

### Data quality control

Data quality was assured through triangulation of semistructured questionnaires, structured observation checklists and training of observers, facilitators, note takers and voice recorders. Data saturation marked sample adequacy. Data was checked for completeness and consistency after completion of the observation.

Efforts were made to minimize the effect of observation on provider behaviors (i.e., the Hawthorne effect [10]) by assuring providers that data collection was anonymous and individual performance would not be reported or shared publicly. Providers were not informed about topics and items of the checklists, so they could not prepare in any way. Observers did not visit facilities where they currently or previously worked as clinicians to minimize the effect of personal and professional relationships.

### Data processing and analysis

The data was analyzed using inductive content analysis [11]. The analysis was started by importing the translated text into open code software version 3.6. Units of relevant meaning were coded sentence by sentence. The investigators gave a code and assigned the emerged categories independently. Discrepancies between coding and category were discussed and negotiated by investigators. The data was related to the research objectives. All categories had similar meanings illustrated by a theme. A single theme represents the overall interpretation of the study and reflects the latent meaning of the data (Table 2).

### Ethical considerations

Ethical approval was obtained from Debre Markos University ethical review committee. A permission and support letter was written to health institutions. Verbal consent was obtained from participants. Confidentiality of information was maintained by omitting any personal identifier from the checklist.

## Results

Forty-three observations were done when the health care providers attended labor. Observations were conducted by using trained bachelor midwives. Maternal blood pressure, maternal pulse and temperature were 13/43, 10/43 and 6/43, respectively (Table 1).

**Table 1:** Observation on partograph utilization at public health facilities in East Gojjam Zone, Northwest Ethiopia, 2015.

Parameters	Yes n = 43

**Progress of labor**	

Check and plot cervical dilation every 4 hours	30
Check and plot descent of head	27
Check and plot uterine contraction every ten minutes	25
**Fetal condition**	

Monitor and plot fetal heart rate every 30 minutes	28
Check and record color of liquor during every per vaginal examination	30
Check and plot molding of fetal skull	19
**Maternal condition**	

Monitor and plot maternal pulse rate every 30 minutes	10
Monitor and plot maternal blood pressure every 4 hours	13
Monitor and plot maternal temperature every 2 hours	6
Monitor and record urine volume, urine protein and ketone every 2 to 4 hours	1

A single independent theme was identified on the whole discussion. Informants’ ideas were put in direct quotation.

### Work overload

Shortage of staff was one of the perceived barriers for the use of the partograph. They said, “There is a shortage of midwives compared to patient flow. Especially during the night shift, the number of cases is high; therefore, one midwife can be conducting two or more labors simultaneously.” Respondents 1, 2, 3, 4, 5 and 6 from FGD2 (see Table 2).

**Table 2:** Theme, categories and codes identified from qualitative data related to perceived barriers to partograph usage during labor management.

Theme Perceived barriers					

Categories	**1-Work overload**	**2-Skill**	**3-Negligence**	**4-Setting**	**5-Misperception**
Code	Lack of time	Lack of Training	Careless	Shortage of medical supply	Partograph is a difficult tool to use
	Shortage of midwives	Lack of awareness	Exhausted	Malfunctioning medical equipment	Partograph is designed for primary care units
	Increased patient flow	Lack of experience	Lack of commitment	Inadequate number of beds and rooms	Misperception
	Lack of motivation	Incompetency	Lack of patience	Frustration and psychologically affected	Believe nothing happened

This reflection revealed there is a midwife shortage in the facility. This could cause ineffective and inefficient use of the partograph during labor because there is no time to attend all laboring mothers using the partograph without rushing. In most health centers, there are only one or two midwives. During night duty, probably one midwife and one clinical nurse can be assigned for all tasks.

Another informant said, “Because of a lack of time and a shortage of midwives, we give more attention to complications … such as bleeding, PROM…. We had never used this tool.” Respondent 2 from FGD1, respondent 1, 4 and 7 from FGD 3 (see Table 2).

Observational checklists showed that most midwives/nurses were not using this tool according to the recommended standard. More than half of midwives were not using the partograph while they attended the labor.

### Skill

Informants explained that skill incompetency and knowledge gaps were barriers to utilizing the partograph. In addition, they explained there was little training about the partograph and its use.

A majority of informants explained that they were not aware of routine checks of urine volume, urine ketone and urine proteins and that these were done for only eclamptic and preeclamptic mothers.

### Negligence

Informants explained the main reason obstetric caregivers utilized the partograph below the required standard was due to negligence. Informants said some of the care providers were careless in utilizing and completing the partograph. We understood that at midnight health caregivers became exhausted and bored. They believed that lack of incentives, recognition, promotion, refreshment, patience and commitment were the main reasons for poor utilization.

Others explained that “although the partograph is a standardized tool used to improve maternal and neonatal outcomes, most midwives and gynecologists are not using this tool consistently.” Respondent 2 from FGD2 (see Table 2).

### Setting

Informants explained there was a lack of delivery rooms, a lack of beds and the rooms are narrow. They explained there were more laboring mothers than beds. They also explained there was a shortage of medical supplies to monitor maternal and fetal conditions, such as thermometers, blood pressure cuffs and urine dipsticks.

Informants said, “The labor and delivery rooms are few and overcrowded; sometimes we attend labors in hospital hallways. Consequently, the mother can be frustrated and affected psychologically, and this can also cause prolonged and obstructed labor, thus unnecessary interventions can be taken.” Respondent 2, 6 FGD 2 (see Table 2).

### Misperception

The respondents explained that some health care providers misunderstand how to use the partograph. One informant said that the “partograph is designed for primary health care units. I believed that the tool is not useful for referral hospitals or tertiary care units.” Respondent 3 FGD 2 (see Table 2).

Informants understood that the ketone, protein and urine volume parts of the partograph were used when the mothers had preeclampsia or eclampsia. One informant declared, “In my opinion, the routine measurement of urine volume, protein and ketone are not advantageous … as I believe these are not vital for normal labor.”

## Discussion

Informants believed that using the partograph during labor and delivery prevented maternal and fetal mortality and morbidity by reducing prolonged and obstructed labor and postpartum hemorrhage (PPH). Consistent with this, studies showed that prolonged and obstructed labor and delayed decisionmaking were the major causes of maternal and fetal death [2,12,13,14], including severe bleeding, infections, hypertensive disorders in pregnancy and obstructed labor. Other studies revealed that partograph utilization had a positive impact on maternal and neonatal delivery-related outcomes [15] and reduced emergency cesarean section rate from 44% to 21% [1,16].

Less than half of care providers were not following WHO recommended standards for maternal and fetal conditions, such as molding (19/43), maternal pulse (10/43), maternal blood pressure (13/43), maternal temperature (6/43) and urine volume, urine protein and urine ketone (1/43). This is supported by qualitative data showing that shortage of staff, lack of awareness, lack of skill and competency, knowledge gaps, lack of commitment, negligence and misunderstanding were the main reasons that participants were not using the partograph routinely. A study done by Dangal G showed that prolonged labor, augmented labor, operative interventions, neonatal morbidity and intrapartum fetal deaths were reduced with the use of the partograph [14,17].

Observation data of the present study showed that the partograph was used below the recommended standard. This is also supported by qualitative data as informants indicated that the partograph was not used properly according to WHO standards. This is comparable to studies conducted in Analamanga, Madagascar [15], and Kenya [3] and studies conducted by Asibong U et al. [18], Opiah MM et al. [19], Julio El-C R et al. [15] Mathibe-Neke J et al. [20] showed that the partograph was used below the suggested standard. Another study done in Tanzania revealed that maternal pulse and blood pressure were used below the standard, 35% and 61%, respectively [21].

This study shows that a shortage of staff, lack of training, lack of skills and little or no knowledge of the partograph were constraints leading to an inability to use the partograph as required, thus compromising effective labor. A similar study conducted in Malawi showed that shortage of staff, negligence, not appreciating the importance of the partograph, ineffective and inadequate supervision, lack of recognition/motivation and skill incompetency were the major contributing factors for underutilization of the partograph [22]. Other studies showed health care providers had low awareness of the partograph [5,23] and skill and knowledge deficits in maternity care [3,4,23,24] were barriers for the use of the partograph. Health providers attitudes and limited confidence [24,25], variation in their commitment [26] and poor interaction with the delivering mother [27] were also barriers. On the other hand, organizational management systems were related to the underutilization of the partograph [7]. Monitoring labor progress using other tools or in addition to the partograph results in poor record keeping and complete documentation of the partograph [4,28].

This study showed that understaffing and high workload, lack of motivation, lack of skills, misperceptions and lack of training were barriers to utilization of the partograph. In line with this, studies conducted in Kenya [3], Ethiopia [8], Uganda [24], South Africa [29], central Nigeria [30] and Mozambique [27] showed understaffing, high workload, frequent staff rotation and job dissatisfaction, lack of knowledge, negative attitudes, medical doctors’ work, and lack of training were barriers to the use of the partograph. In Central Ethiopia, use of different monitoring tools, a shortage of staff and a lack of trained caregiver were the reasons for not using the partograph during labor [9].

## Conclusion

The overall assessment revealed that the partograph was not used up to the recommended standard. Informants explained that proper use of the partograph could reduce maternal and fetal adverse outcomes. They believed that it could also prevent prolonged and obstructed labor, fistula and bleeding. The main reasons for underutilization of the partograph were lack of awareness, skill inconsistency, lack of commitment, lack of motivation, work overload, unfavorable attitudes and a shortage of resources and infrastructure. Little is known about the challenges of using the partograph. Detailed understanding of these challenges will be important to inform policy makers, stakeholders, program planners and obstetric caregivers in order to develop appropriate policies and strategies to improve the quality of intrapartum care.
